# The efficacy of traditional Chinese medicine in the treatment of the COVID-19 pandemic in Henan Province: a retrospective study

**DOI:** 10.1186/s40001-023-01006-9

**Published:** 2023-02-13

**Authors:** Ruiting Han, Yang Xie, Hulei Zhao, Bin Li, Xueqing Yu, Minghang Wang, Suyun Li, Jiansheng Li

**Affiliations:** 1 Department of Respiratory Diseases, The First Affiliated Hospital of Henan University of Chinese Medicine, Zhengzhou, 450000 Henan China; 2 Collaborative Innovation Center for Chinese Medicine and Respiratory Diseases Co-Constructed By Henan Province & Education Ministry of P.R. China, Henan University of Chinese Medicine, Zhengzhou, 450046 Henan China

**Keywords:** COVID-19, Chinese patent medicines, Chinese medicine injections, Long of stay, Traditional Chinese medicine

## Abstract

**Background:**

Since 2020, novel coronavirus disease (COVID-19) has posed serious threats to health systems and led to tremendous economic decline worldwide. Traditional Chinese medicine (TCM) is considered a promising treatment strategy for COVID-19 in China and is increasingly recognized as a key participant in the battle against COVID-19. Clinicians also need accurate evidence regarding the effectiveness of TCM treatments for COVID-19.

**Methods:**

We retrospectively analyzed patients diagnosed with COVID-19 by collected from the electronic medical records of the hospitals in Henan Province from January 19, 2020, to March 2, 2020. Demographic characteristics, clinical data, frequency analysis of Chinese patent medicines (CPMs), Chinese medicine injections (CMIs), evaluation of baseline symptom scores, nucleic acid negative conversion, length of hospitalization, and mortality rates were studied.

**Results:**

Between 15 January 2020 and 2 March 2020, 131 hospitals with 1245 patients were included. Survey response Chinese herbal decoction, CPMs, and CMIs combined with conventional Western medicine (CWM) used for the treatment of COVID-19. The top 8 CPMs were Lianhua Qingwen capsules, Shuanghuanglian oral liquid, Pudilan Xiaoyan oral liquid, Banlangen granules, Lanqin oral liquid, compound licorice tablets, Bailing capsules, montmorillonite powder. The most frequently used CMIs were Xuebijing, Tanreqing, Reduning, Xiyanping and Yanhuning. TCM combined with CWM improved the patients’ symptom scores for fever, cough, chest tightness, shortness of breath, and fatigue. Nucleic acid negative conversion occurred at11.55 ± 5.91 d and the average length of hospitalization was 14.92 ± 6.15 d. The mortality rate was approximately 1.76%, which is a reduction in patient mortality.

**Conclusions:**

TCM combined with CWM improved clinical symptoms and reduced hospitalization and mortality rates.

## Introduction

COVID-19 has remarkably weakened the global economy by threatening health systems worldwide. Recently, there have been 643,875,406 confirmed cases of COVID-19, including 6,630,082 deaths, a total of 12,998,974,878 vaccine doses have been administered, all over 200 countries and territories (WHO, https://covid19.who.int/, as of 4 December 2022), however, the long-term outcomes of the COVID-19 pandemic are still difficult to predict. Thus far, effective drugs to treat COVID-19 are lacking, hence, it is necessary to explore several potential antiviral drug strategies for COVID-19, especially complementary and integrative medicines.

Traditional Chinese medicine (TCM) is promising for the treatment and prevention of COVID-19, and it is expected that TCM will be promoted by countries worldwide [1]. Numerous studies have reported that combined therapy with TCM and conventional Western medicine (CWM) significantly reduced mortality and improved fever and cough symptoms compared to using CWM alone [2]. Studies have shown that using TCM to treat COVID-19 in mainland China has brought new hope to the fight against the pandemic and has also provided many potentially effective drugs for further optimization [3].

In this study, we extracted a series of interrelated data, including epidemiological information, TCM syndrome differentiation, clinical characteristics, as well as data on the effectiveness and safety of integrated TCM and CWM in COVID-19 patients in Henan Province. We aim to explore the association of utilizations of TCM in the treatment of COVID-19, and clinical outcomes included in-hospital mortality, length of hospitalization. We present evidence for the diagnosis and treatment of COVID-19, as well as a brief overview of the status of and issues with TCM in the treatment of this global pandemic.

## Methods

### Data source and study sample

In the retrospective study of patients who were hospitalized with laboratory-confirmed SARS-CoV-2 infections in 131 hospitals, which were involved in epidemic prevention and control between 15 January 2020 and 2 March 2020 in Henan Province. (The First Affiliated Hospital of Henan University of CM, The First Affiliated Hospital of Zhengzhou University, Henan Provincial People’s Hospital, The Sixth Peoples Hospital of Zhengzhou, Nanyang Central Hospital, Zhumadian Central Hospital, The First Affiliated Hospital of Henan University of Science and Technology, Fifth People's Hospital of Anyang, among others). Electronic medical records (EMRs) were extracted and a data form was used to collect patient characteristics, such as patients’ gender, age, Wuhan or Hubei travel history, comorbidities, treatment, and clinical outcomes.

In terms of data quality control, owing to the variety and dosage forms of TCM, the name of the drug is standardized and different dosage forms of the same drug are standardized as one. Two researchers independently verified the eligibility of the patients for this study and retrieved the data.

### COVID-19 diagnosis and treatment protocol

Patients were treated in accordance with the Diagnosis and Treatment Protocol for Novel Coronavirus Pneumonia (5th version) published by the National Health Commission of China. The treatment strategies for COVID-19 include CWM combined with TCM, CWM including oxygen therapy, antibiotics, antiviral medications, nutritional support, and other conventional treatments. TCM is used in a variety of dosage forms, including Chinese herbal decoctions, Chinese patent medicines (CPMs), granules, pills, Chinese medicine injections (CMIs) and other interventions.

### Outcome assessments

#### Demographic characteristics and clinical symptoms

Demographic characteristics of patients with COVID-19, including sex, age, marital status, travel history, and comorbidities. We also assessed the clinical symptoms, including body temperature, cough, shortness of breath, chest tightness in severe cases, and fatigue at baseline and at 4, 7, 10, and 14 d after treatment.

#### Nucleic acid negative conversion, length of hospitalization and mortality rate

The gold standard for the diagnosis of COVID-19 is by RT-PCR of nasal or throat swabs. The first positive test was set as the date of diagnosis, and the date of two consecutive negative PCR tests was used as the cutoff, to calculate the length of the hospital stay. Mortality during hospitalization was also extracted.

### Statistical analysis

Descriptive statistics were assessed as frequency, composition ratio or percentage. Continuous variables were expressed as mean and standard deviation (SD). For statistical analysis was performed using the Kruskal Wallis, results was considered statistically significant if the *p* value < 0.05. Data were analyzed using Origin software (version 2022).

## Results

### Population characteristics

**Fig. 1:**
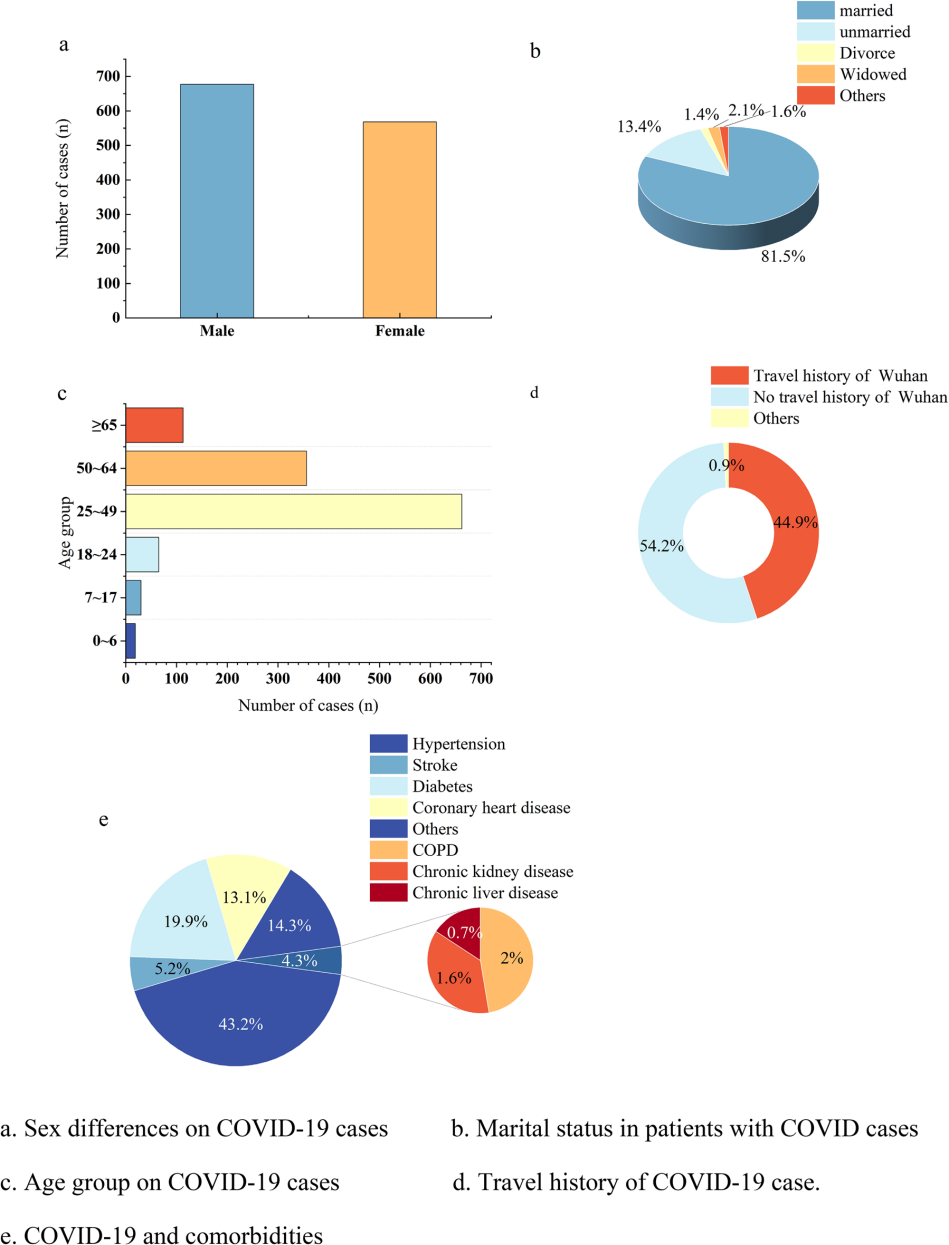
Demographic characteristics of COVID-19 in Henan Province

A total of 1245 cases with confirmed COVID-19 infection were analyzed. Among the patients, 677 (54.3%) were male and 568 (45.6%) were female, of the patients 1015 were married (approximately 81%). Infections occurred in patients of all ages, including newborn infants and the elderly, with high incidences of the disease at 25–49 (662) and 50–64 years (356). A total of 559 (44.8%) patients had a history of travel or residence in Wuhan, and 54.2% (675/1245) had a history of travel or residence in Henan Provinces other than Wuhan or Hubei. Comorbidities were common, the most common comorbidities were hypertension (191), diabetes (88), stroke (23), coronary heart disease (58), chronic kidney disease (8), chronic obstructive pulmonary disease (7), Chronic liver disease (3), and other abnormalities (63). The demographic characteristics of the participants are presented in Fig. 1.

### TCM syndrome characteristics

The clinical symptom data of the patients at different admission times (baseline), after 4, 7, 10 and 14 days of hospitalisation, were summarised. Symptom scores, particularly respiratory symptoms at each timepoint, were compared with baseline, including fever, cough, chest tightness, shortness of breath and fatigue. The symptom scores of the different nodes before and after treatment were used to assess the improvement of the patients' symptoms.

### Chinese herbal decoction treatment for COVID-19

Chinese herbal decoctions with good efficacy against COVID-19, including active pharmaceutical ingredients, for different stages of COVID-19 treatment (quercetin, wogonin, beta-sitosterol, baicalein, Kaempferol, luteolin, etc.). Various Chinese herbal formulations, such as Jing Fang Bai Du San or Yinqiao powder, have been used in the mild stage, when cold‒damp constrain and damp‒heat accumulation in the lung. The main patterns in the middle stage were damp‒toxin constraint in the lung and cold‒damp obstructing the lung; therefore, medicines such as Huopo xialing decoction, Mahuang dingchuan decoction, Ganlu xiaodu Dan, and Huashi baidu Fang were used to eliminate dampness. Huoxiang zhengqi is a commonly used damp clearing prescription that has the efficacy of reliving superficies, dispersing dampness, and harmonizing stomach. Later in the recovery period, Shasen Maidong was used to revitalize Qi and Yin, and Si/Liu junzi decoctions were used to nourish Qi and strengthen the spleen. These herbal decoctions contain a variety of components, including flavonoids, alkaloids, terpenoids, and polyphenols, and can alleviating cytokine storms, regulate immune imbalances, and produce the potential effect of synergistic treatment for COVID-19 quickly and effectively (Table 1).

**Table 1:** Pharmacological effects of Chinese herbal decoction and ingredients against COVID-19

TCM Syndrome differentiation	Formula	Active ingredients	Pharmacological effects of Formula against COVID-19
Cold‒damp constraint	Jing Fang Bai Du San	Acacetin, glypallichalcone, wogonin, gancaonin A, isorhamnetin, etc	Inhibits the virus' entry into host cells and its replication
Damp‒heat accumulation in the lung	Yinqiao powder	Hesperetin, Eriodictyol, Luteolin, Quercetin, Naringenin, etc	Inhibit inflammatory responses by suppressing IL-6, CXCL2, TNF*α*, NF-*κ*B, etc
	Sangbaipi	Gardenoside, rutin, berberine, palmatine, baicalein, wogonin, etc	Exert anti-inflammatory effect
Damp‒toxin constraint in the lung and cold‒damp obstructing the lung	Huopo xialing	Baicalein, beta-sitosterol, irisolidone, quercetin, etc	Intervening inflammatory response, immune regulation and apoptosis
	Ganluxiaodu Dan	Quercetin, beta-sitosterol, wogonin, irisolidone, kaempferol, etc	Regulate interfere with SARS-CoV-2 infection through antivirus, inhibition of inflammatory factors and regulation of immunity
	Huashi baidu granule	Quercetin, kaempferol, luteolin, wogonin, 7-o-methylisomucronulatol, naringenin, baicalein, beta-sitosterol, etc	Antivirus, anti-inflammation
Dampness obstructing the lung and stomach	Huoxiang zhengqi	Quercetin, isorhamnetin, irisolidone, kaempferol, wogonin, baicalein, etc	Antivirus, anti-inflammation and relieving symptoms
Deficiency of Qi and Yin	Shasen Maidong	Quercetin, Beta-carotene, Stigmasterol, Kaempferol, beta-sitosterol, Naringenin	Anti-inflammatory, improving immunity
Qi-deficiency of lung and spleen	Si/Liu junzi	Mairin, naringenin, euchrenone, glepidotinA, sigmoidin-B, icos-5-enoic acid, gadelaidic acid, xambioona, trametenolic acid, 3pachymic acid, poricoic acid C, hederagenin, dehydroeburicoic acid, chondrillasterol, spinasterol, stigmast-7-enol , etc	Regulate immunity, reduce lung injury, promote cell growth and differentiation and pulmonary angiogenesis,

### Chinese patent medicines for COVID-19

**Fig. 2:**
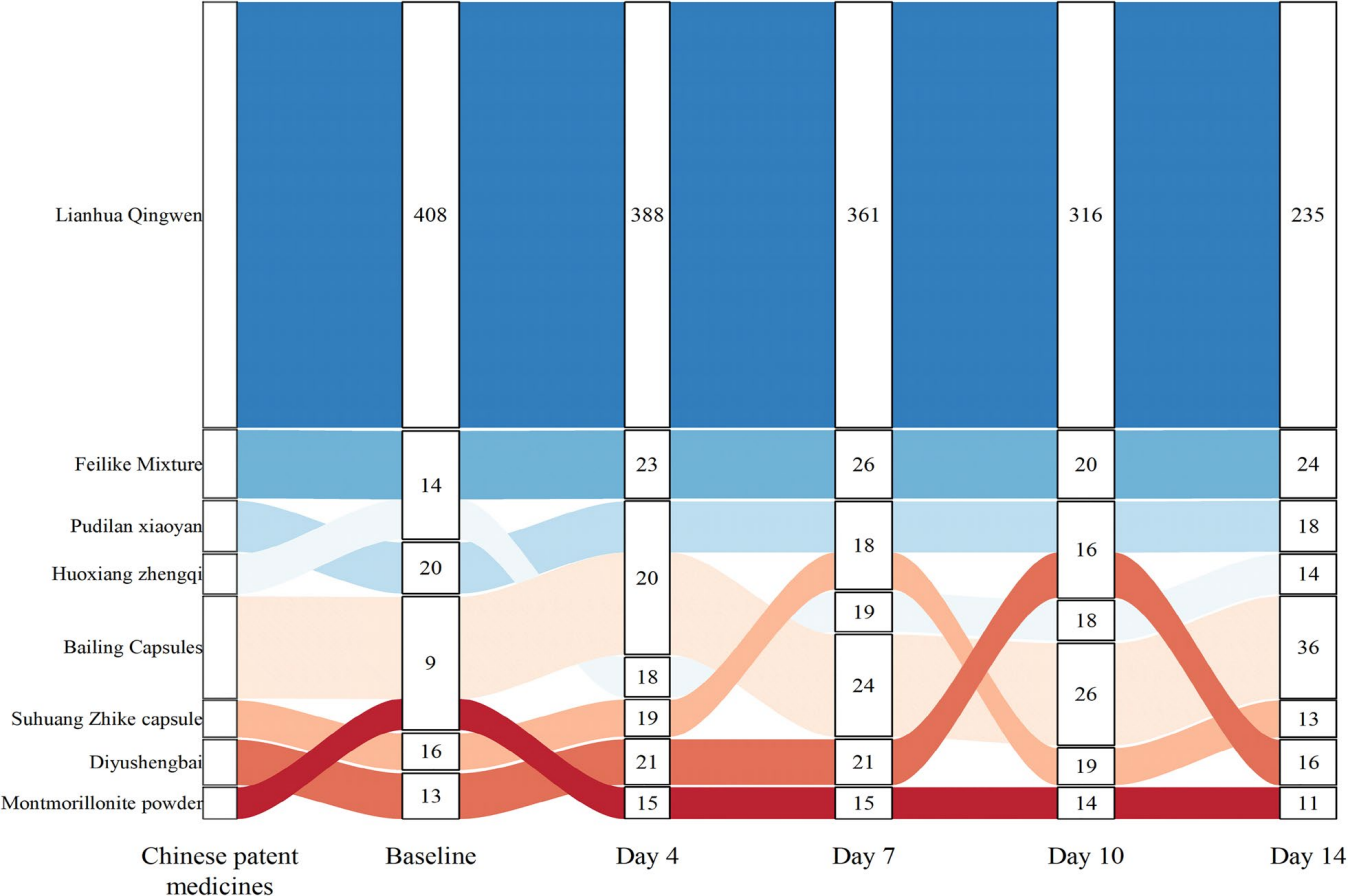
Top 8 CPMs in the 5 timepoints used for COVID-19 (Frequency ≥ 8). The number of patients who utilize a particular Chinese medication at the designated time period is the definition of "frequency."

According to our findings, the applications of Chinese patent medicines (CPMs) fall into four categories: heat-clearing agents, dampness-removing agents, cough suppressants, and tonics. Lianhua Qingwen, Shuanghuanglian Oral Liquid, and Pudilan Xiaoyan oral liquid are examples of representative heat-clearing and detoxifying CPMs. Damp-removing agents include Huoxiang zhengqi soft capsules, which have a similar pharmacological effect to decoction. Cough medications include Feilike mixture, Suhuang Zhike capsules, and Xuanfei Zhike mixture. Tonics such as Bailing capsules and Diyushengbai improve immune function. When the use of CPMs at 5 timepoints was summarized, Lianhua Qingwen was the most frequently used at all timepoints. With the passage of time, the total frequency of treatment drugs is also gradually decreasing; interestingly, Bailing Capsules are being used more frequently, placing them second in the 14 day administration (Fig. 2).

### Chinese medicine injections

**Fig. 3:**
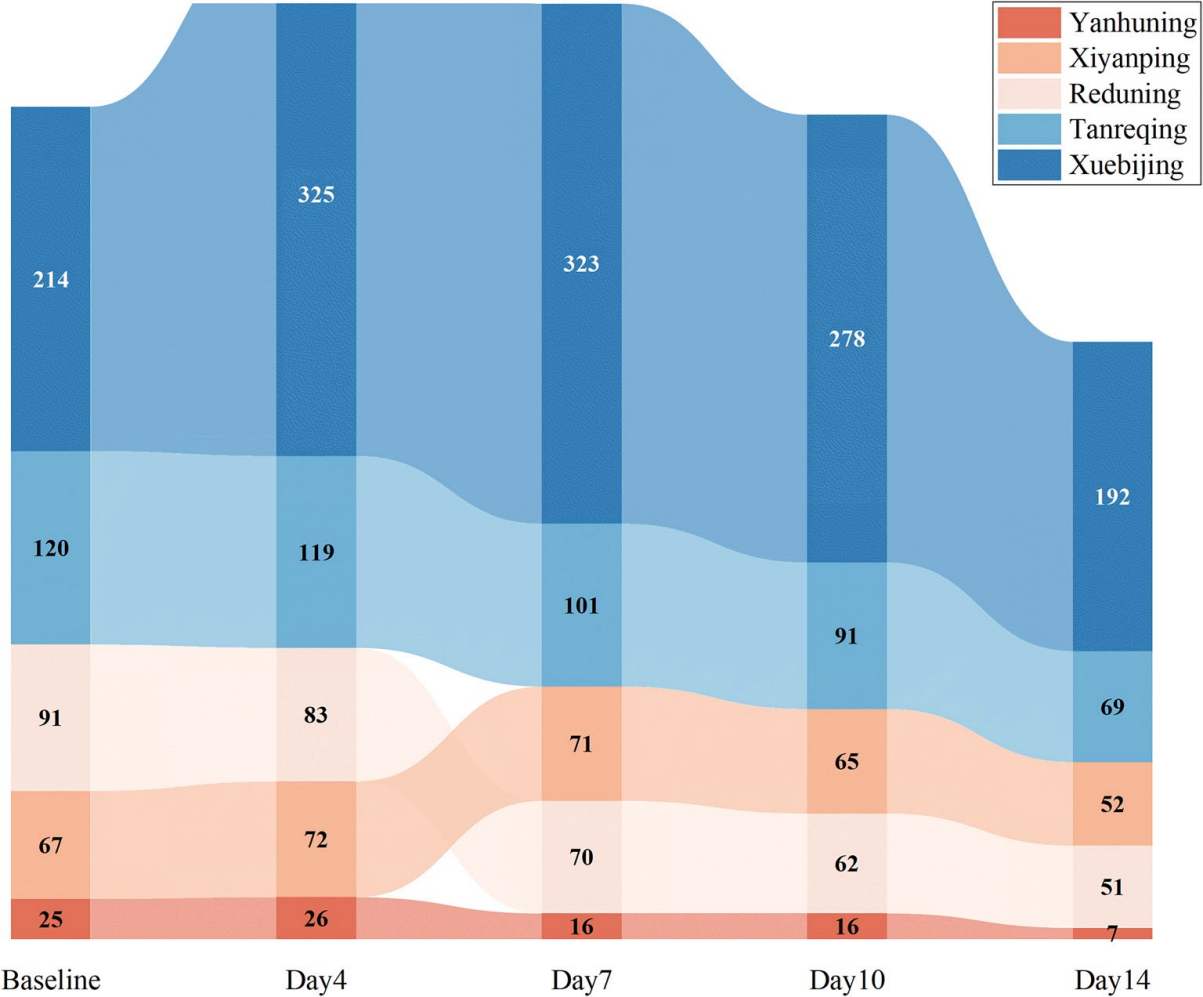
Top 5 CMI in the 5 timepoints used for COVID-19 (Frequently ≥ 6)

Chinese medicine injections (CMIs) have become more widespread in China because of their instantaneous effects, fewer side effects, and significant tonic function. Furthermore, CMIs have been suggested to be significantly effective not only in the digestive and cardiovascular systems, but also in the respiratory system. We found that from admission to 4, 7, and 10 d of hospitalization, the most frequently used CMIs were Xuebijing, Tanreqing, Reduning, Xiyanping and Yanhuning, Similarly, the total frequency of CMIs is gradually decreasing over time, which is consistent with the trend of CPMs use (Fig. 3).

### Clinical efficacy assessment

**Fig. 4:**
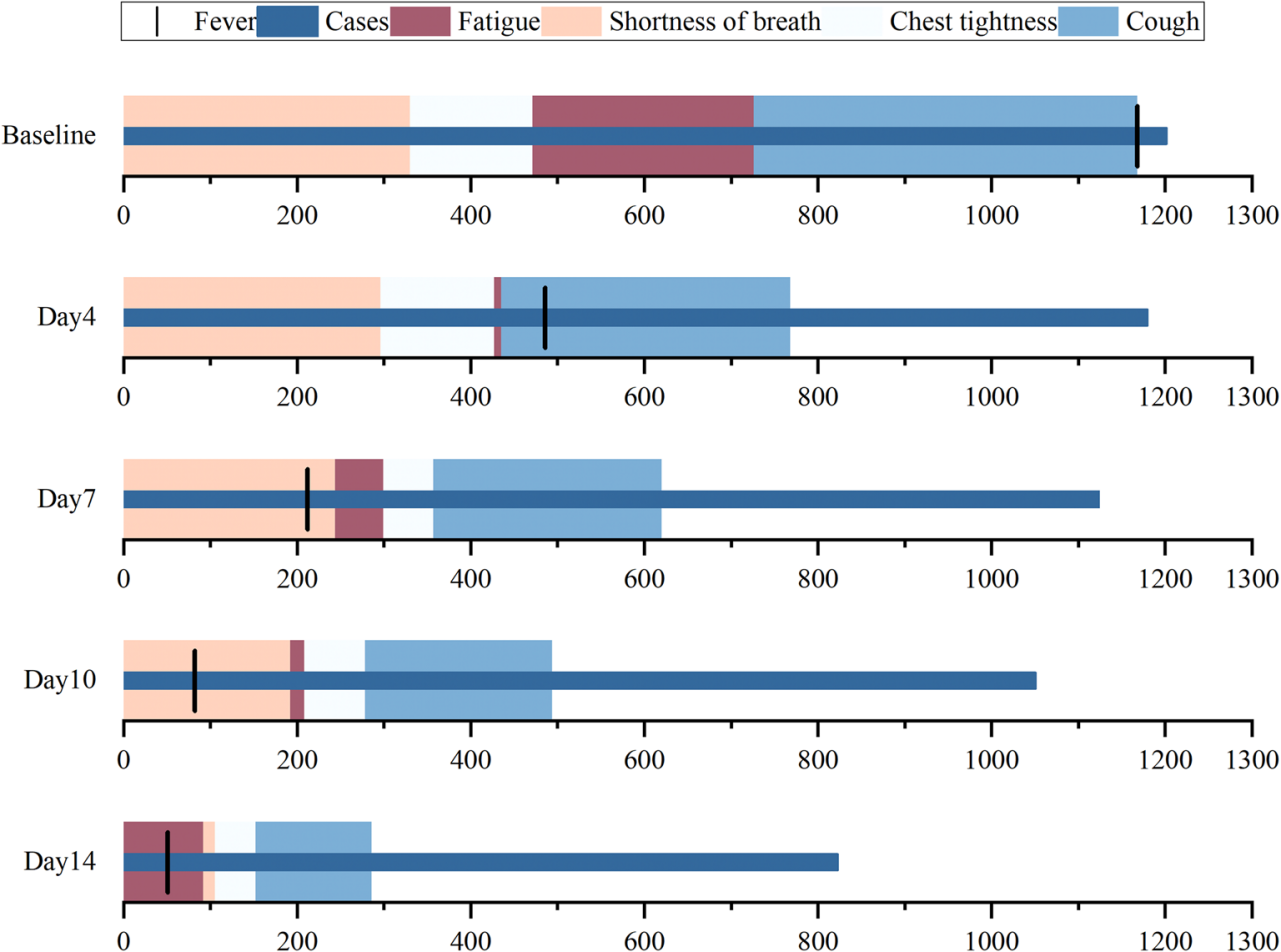
Clinical symptom scores at different timepoints

COVID-19 predominantly presents as a respiratory infection, with clinical manifestations including fever, cough, chest tightness, shortness of breath in severe cases, and fatigue. Patient clinical symptom information was used to evaluate the curative effect. We compared the clinical signs scored and plotted them at four timepoints, as indicated. Fever, fatigue and cough symptom scores significantly lower at days 4, 7, 10, and 14 compared to baseline (*p* < 0.05), chest tightness scores were significantly lower than baseline at days 10 and 14 after treatment, shortness of breath was statistically significant only after 14 days of treatment (*p* < 0.05). With the treatment stage, some patients are discharged from the hospital, and the number of patients and symptom scores are gradually decreasing (Fig. 4).

### Nucleic acid negative conversion, length of hospital stay, and mortality rate

**Fig. 5:**
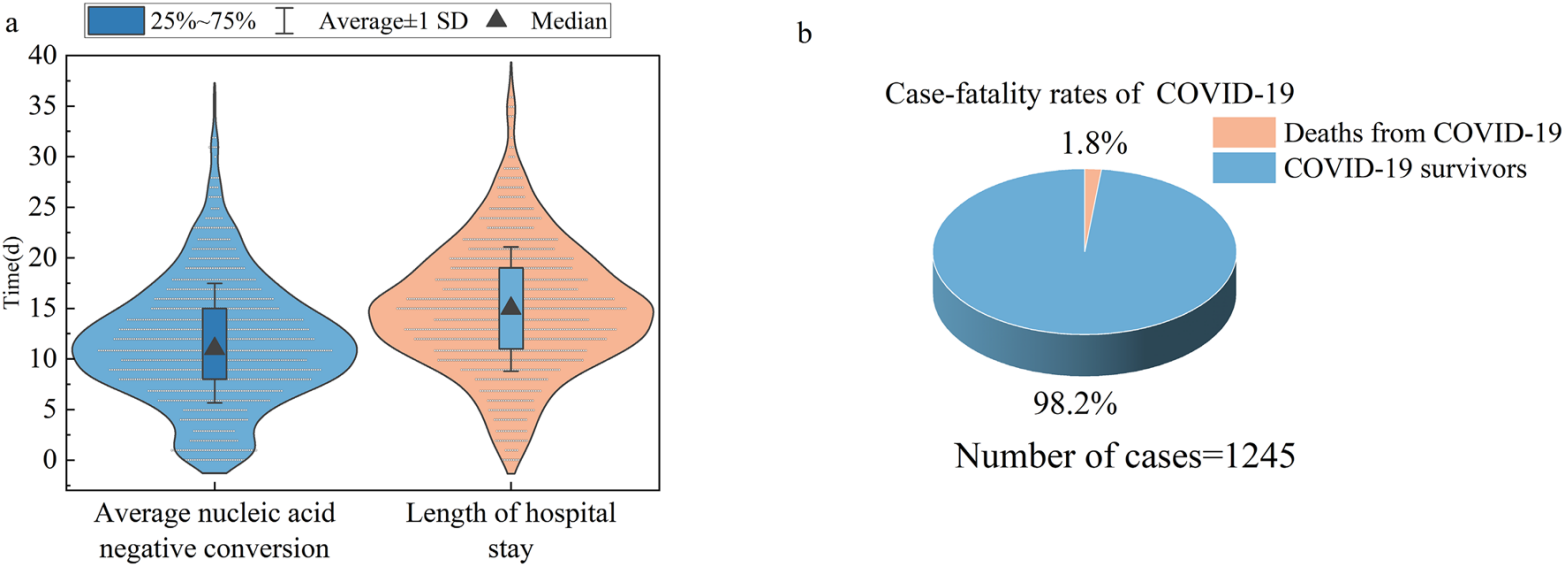
Nucleic acid negative conversion, length of hospital stay, and mortality rate

There were 1245 patients with a complete nucleic acid negative conversion date, with an average nucleic acid negative conversion of 11.55 ± 5.91 days and their average hospital stay was 14.92 ± 6.15 days (Fig. 5a). In our study, there were 22 fatal cases before February 29, 2020, and their average age was 71.2 years. The mortality rate was 1.76% (22/1245). Among the fatalities, 17 cases had well-defined complex comorbidities, including hypertension, chronic obstructive pulmonary disease, diabetes, and severe obesity, which were the most common comorbidities in the non-survivors (Fig. 5b).

## Discussion

COVID-19 belongs to the “epidemic disease” category in TCM. COVID-19 is spread primarily via respiratory droplets during close face-to-face contact, which is caused by the “yi qi” feeling of the disease. COVID-19 patients in Henan Province are mainly 18–60 years, with greater prevalence among men than women. Most patients had normal symptoms, and those who were severely or critically ill patients had underlying diseases, including hypertension, coronary heart disease, and diabetes. The complexity of comorbidities affects disease progression and patient prognosis. In our study, we found that 45% of the reported cases had a history of residence or travel in Wuhan.

Most patients with COVID-19 present with respiratory symptoms, such as fever, cough, chest tightness, and fatigue. In addition, patients exhibit a wide range of clinical manifestations, from asymptomatic to symptomatic. Previous studies have noted that a small percentage of patients experience gastrointestinal symptoms, with the most common being vomiting, abdominal pain, and diarrhea. A strong relationship between COVID-19 and the gut microbiome has been reported in literature [4]. Based on the theory of syndrome differentiation and the holistic view of TCM, it can simultaneously treat respiratory and gastrointestinal symptoms in COVID-19 patients.

A classified analysis of the efficacy and advantages of TCM for the prevention and treatment of COVID-19 has been performed, and this study can be a valuable reference for the application of TCM. Several representative Chinese herbal medicine formulations, including Jing Fang Bai Du San, were used to treat prevalent epidemic pathogens in ancient China. Jing Fang Bai Du San was found to temporarily improve the clinical symptoms of patients, and network pharmacology analysis initially revealed that the targets of Jing Fang Bai Du San in the treatment of COVID-19 mainly included EGFR, PIK3CA, LCK, and MAPK1 [5].

CPMs have been invaluable in the treatment of COVID-19. One of the most important TCM principles in the treatment of COVID-19 is syndrome differentiation. CPMs have the advantages of convenience and speed, and they can be used dialectically in the treatment of COVID-19 at various stages of the disease. The top ten CPMs have antiviral, antibacterial, and anti-inflammatory properties, as well as the ability to regulate immune inflammatory responses, protect against organ damage, improve pulmonary function, and alleviate clinical symptoms. A study found that LHQW can prevent or delay the progression to severe COVID-19 and inhibit influenza-induced bacterial adhesion by inhibiting adhesion molecules and improving severe pneumonia [6, 7]. HXZQ not only alleviates intestinal damage, but it also has anti-inflammatory and immunomodulatory properties in COVID-19, inhibiting inflammatory factors and modulating immune responses [8]. A randomized controlled trial discovered that HXZQ dropping pills and LHQW granules combined with CWM may have clinical benefits for improving clinical symptoms of COVID-19 patients, lowering anti-infective drug utilization, and improving patient prognosis [9]. FLKM has been shown in animal studies to alleviate pathological lung tissue injury, reduce neutrophil infiltration in rats with LPS-induced pneumonia, and alleviate clinical symptoms and signs in patients with pneumonia [10]. PDL protects against LPS-induced respiratory inflammation in mice [11]. Upper respiratory symptoms, fever, and altered taste remained the most common presenting symptoms. Extrapulmonary complications are also common, including the gastrointestinal tract, which explains the frequent use of Huoxiangzhengqi and montmorillonite.

CMIs significantly reduce the clinical symptoms of patients and prevent COVID-19 complications. CMIs were first recommended by the Chinese Clinical Guidance of COVID-19 Pneumonia Diagnosis and Treatment (4th edition), which suggested the application of Xiyanping injection in medium-term clinical treatment [12]. The results revealed that Xuebijing, Tanreqing, Reduning, Xiyanping, and Yanhuning were the most frequently used CMIs for COVID-19. Studies have found that Xuebijing has significant curative effects on sepsis through anti-inflammatory, anticoagulatory, immunoregulatory, vascular endothelial protective, and anti-oxidative stress effects. Tanreqing injection combined with CWM can effectively shorten the fever reduction time of community-acquired pneumonia and the hospitalization time of patients [13]. In vivo experiments have shown that Tanreqing injection can notably inhibit the expression of TNF-α, IL-6, IL-8, and IL-17A at both the gene and protein levels [14]. Reduning injection has achieved a certain effect in the clinical treatment of acute bronchitis and upper respiratory tract infections [15]. A multicenter, prospective, open-label, and randomized controlled trial on Xiyanping injection found that it can remarkably shorten the period of coughing and improve the recovery of patients with mild to moderate COVID-19 [16].

A series of CPMs and CMIs have played a promising role in the treatment of COVID-19.The most obvious finding to emerge from the analysis was that the symptoms of cough, fever, chest tightness, shortness of breath, fatigue, and other symptoms of the patients were significantly improved after a series of treatments. The average length of hospital stay (LOS) of patients in this study period was 14.92 ± 6.15 d, and the median LOS in the hospital was 14 d, with a range of 2–46 days. This may because most cases in Henan Province are mild and with common symptoms. In addition, we found that the case fatality rate (CFR) of patients with COVID-19 was approximately 1.76%, which is close to 2%. One study on 172 patients with COVID-19 between January 2020 and February 2020 in Henan Province had a CFR of 2.3% [17]. These results are in accordance with personal data from COVID-19 cases detected outside mainland China, which had an average CFR of 3.6%. [18]. A systematic review and meta-analysis revealed that the CFR of COVID-19 was 10%, while in hospitalized patients, the CFR was 13.0%. Among ICU patients, the CFR was as high as 37% [19]. The reason for the consistently low fatality rate in Henan Province may be closely related to the effects of TCM, and there may also be significant associations with patient age, sex, comorbidities, and stage after infection. In our study, we found that TCM with CWM has unique advantages in improving clinical symptoms, improving cure rate, reducing hospital stay, delaying disease progression, and reducing mortality.

## Limitations

Despite these promising results, this study has some limitations. First, the progress of COVID-19 treatment was not analyzed and there was a lack of a randomized control group. Second, some of the electronic medical records were incomplete. In addition, the combined application of different dosages of Chinese herbs have many complex ingredients, and their mechanisms of action need to be explored further. Despite the persistent limitations of TCM in epidemiological investigations, this study provides supplementary data on the clinical course of COVID-19 when TCM was used. Further studies should focus on improving the safety and efficacy of Chinese medicines to treat this respiratory epidemic.

## Conclusion

In conclusion, our presentation provides better options and more convincing evidence for the clinical treatment of COVID-19. TCM combined with CWM improved clinical symptoms and reduced hospitalization and mortality rates. TCM has been practiced for thousands of years to treat large-scale pandemic diseases, and we hope that TCM can considerably control this novel respiratory infectious disease.

## Data Availability

The data sets from this study are available from the first author upon request.

## Abbreviations

CFR: Case fatality rate
CMIs: Chinese medicine injections
COVID-19: Coronavirus disease 2019
CPMs: Chinese patent medicines
CWM: Conventional western medicine
EMRs: Electronic medical records
Los: Length of stay
TCM: Traditional Chinese medicine
